# Characterization of the SF3B1–SUGP1 interface reveals how numerous cancer mutations cause mRNA missplicing

**DOI:** 10.1101/gad.351154.123

**Published:** 2023

**Authors:** Jian Zhang, Jindou Xie, Ji Huang, Xiangyang Liu, Ruihong Xu, Jonas Tholen, Wojciech P. Galej, Liang Tong, James L. Manley, Zhaoqi Liu

**Affiliations:** 1 Department of Biological Sciences, Columbia University, New York, New York 10027, USA;; 2 CAS Key Laboratory of Genomic and Precision Medicine, Beijing Institute of Genomics, Chinese Academy of Sciences and China National Center for Bioinformation, Beijing 100101, China;; 3 University of Chinese Academy of Sciences, Beijing 100049, China;; 4 European Molecular Biology Laboratory, 38042 Grenoble, France

**Keywords:** spliceosome mutations, AlphaFold-Multimer, DHX15, myelodysplastic syndromes, G-patch

## Abstract

The spliceosomal gene *SF3B1* is frequently mutated in cancer. While it is known that *SF3B1* hotspot mutations lead to loss of splicing factor SUGP1 from spliceosomes, the cancer-relevant SF3B1–SUGP1 interaction has not been characterized. To address this issue, we show by structural modeling that two regions flanking the SUGP1 G-patch make numerous contacts with the region of SF3B1 harboring hotspot mutations. Experiments confirmed that all the cancer-associated mutations in these regions, as well as mutations affecting other residues in the SF3B1–SUGP1 interface, not only weaken or disrupt the interaction but also alter splicing similarly to *SF3B1* cancer mutations. Finally, structural modeling of a trimeric protein complex reveals that the SF3B1–SUGP1 interaction “loops out” the G-patch for interaction with the helicase DHX15. Our study thus provides an unprecedented molecular view of a protein complex essential for accurate splicing and also reveals that numerous cancer-associated mutations disrupt the critical SF3B1–SUGP1 interaction.

## Abstract (precis)

In this study, Zhang et al. provide molecular and structural insights into the cancer-relevant interaction between spliceosome factors SF3B1 and SUGP1. They further elucidate the effect of cancer-associated mutations in SF3B1 and SUGP1 that weaken their interaction, disrupting the formation of a larger complex with DHX15 and causing pre-mRNA splicing defects.

*SF3B1* (splicing factor 3b subunit 1) is the most frequently mutated spliceosomal gene in hematological malignancies and some solid tumors (Yoshida et al. 2011; Yoshida and Ogawa 2014; Seiler et al. 2018). Recurrent mutations are found in a wide variety of cancers, including myelodysplastic syndromes (Papaemmanuil et al. 2011; Yoshida et al. 2011; Haferlach et al. 2014), chronic lymphocytic leukemia (Wang et al. 2011; Quesada et al. 2012), uveal and mucosal melanomas (Harbour et al. 2013; Hintzsche et al. 2017), and other solid tumors (Biankin et al. 2012; Ellis et al. 2012; Seiler et al. 2018). Despite the important role of mutant SF3B1 in tumorigenesis, the molecular basis by which the numerous *SF3B1* cancer mutations lead to RNA missplicing remains unclear.

SF3B1 is a major component of the spliceosome, a large ribonucleoprotein complex that carries out precursor mRNA (pre-mRNA) splicing. The spliceosome is composed of five small nuclear RNAs (snRNAs) (namely, U1, U2, U4, U5, and U6) and >100 associated proteins (Wahl et al. 2009). Assembly of the spliceosome is highly dynamic and is initiated by binding of U1 and U2 small nuclear ribonucleoproteins (snRNPs) to the splicing signals in the intron: 5′ splice site (ss), 3′ss, polypyrimidine tract, and branch site. After recruitment of the U4/U6.U5 tri-snRNP and structural rearrangements, the fully assembled spliceosome is activated and carries out two sequential transesterification reactions to remove the intron and join together the flanking exons (Wahl et al. 2009). During each splicing cycle, the spliceosome rapidly changes its composition and conformation to carry out its function and is now thought to progress through numerous distinct conformational states, including E, A, pre-B, B, B^act^, B*, C, C*, P, and intron lariat spliceosome (ILS) complexes (Wahl et al. 2009; Rodrigues et al. 2023).

Almost all the multiple cancer mutations in *SF3B1* are located in sequences encoding its C-terminal HEAT (huntingtin, elongation factor 3, A subunit of protein phosphatase 2A, and target of rapamycin 1) domain, which is composed of 20 HEAT repeats (Cretu et al. 2016). The great majority of the mutations, including the most common mutation (K700E), are located in HEAT repeats H4–H7 (Cretu et al. 2016). Hotspot mutations in *SF3B1* induce use of cryptic 3′ splice sites typically located ∼10–30 nt upstream of the associated canonical 3′ splice sites by promoting recognition of alternative branch sites (Darman et al. 2015; DeBoever et al. 2015; Alsafadi et al. 2016; Zhang et al. 2019a), and some of the resulting splicing errors contribute to severe cancer phenotypes (Inoue et al. 2019; Lieu et al. 2022). We previously showed that several common *SF3B1* mutations, including K700E, lead to loss of the splicing factor SUGP1 (SURP and G-patch domain-containing 1) from the mutant spliceosome due to a defective interaction between mutant SF3B1 and SUGP1 (Zhang et al. 2019a). Pan-cancer analyses of >10,000 samples in TCGA also revealed a number of *SUGP1* cancer-associated mutations flanking the SUGP1 G-patch motif (G-patch) that partially recapitulate mutant SF3B1 missplicing, suggesting a mechanistic link between SF3B1 and SUGP1 (Liu et al. 2020; Alsafadi et al. 2021). The G-patch is especially important in mediating SUGP1 function in accurate 3′ss selection (Zhang et al. 2019a) and functions via an activating interaction with DEAH-box helicase 15 (DHX15) (Zhang et al. 2022a; Beusch et al. 2023; Feng et al. 2023). However, how SF3B1 and SUGP1 interact is not known, leaving gaps in our understanding of both how branch sites and 3′ splice sites are correctly selected and how cancer mutations disrupt this process.

There are now multiple cryo-electron microscopy (cryo-EM) structures of human spliceosomal complexes: pre-A, pre-B, B, B^act^, C, C*, P, and ILS, as well as the 17S U2 snRNP (the major subunit of E and A complexes) (Agafonov et al. 2016; Bertram et al. 2017a,b; Zhang et al. 2017, 2018, 2019b, 2020, 2022b; Haselbach et al. 2018; Zhan et al. 2018; Tholen et al. 2022). However, SUGP1 was not observed in any of these stage-specific complexes, suggesting that SUGP1 likely participates in an uncharacterized intermediate complex during branch site recognition and that its interactions are expected to be transient. This fact poses a great challenge to study the SF3B1–SUGP1 interaction in the spliceosome. Predicting the 3D structures of proteins using advanced deep-learning algorithms offers an alternative opportunity to tackle this issue. Specifically, the AlphaFold2 algorithm has achieved unprecedented accuracy of structure prediction for single proteins (Jumper et al. 2021). Its new module, AlphaFold-Multimer, has been specifically refined to predict structures of protein–protein complexes and has achieved 70% and 72% accuracy for predicting heteromeric and homomeric interfaces, respectively (Evans et al. 2022).

In this study, we used computational structural modeling together with experimental analyses to characterize the nature of the SF3B1–SUGP1 interaction and to elucidate the role of cancer-relevant mutations in both *SF3B1* and *SUGP1* in RNA missplicing. AlphaFold-Multimer modeling of the SF3B1–SUGP1 heterodimer structure revealed that two separate regions flanking the SUGP1 G-patch make numerous direct contacts with the region of SF3B1 harboring multiple hotspot cancer mutations. We experimentally confirmed that all the cancer-associated missense mutations in these two regions of SUGP1 weaken or abolish its interaction with SF3B1. We also confirmed that other specific SUGP1 residues located in the SF3B1–SUGP1 interface, including, remarkably, the very last two residues (both tyrosines) at the C terminus, are critical for interaction. Importantly, expression of SUGP1 derivatives containing mutations or deletions of these residues partially recapitulated the RNA splicing defects of mutant SF3B1. The two SF3B1-interacting regions in SUGP1 closely surround, but do not overlap, the SUGP1 G-patch, thereby “looping out” this region for interaction with its target, the helicase DHX15. Our study thus provides a detailed view of a protein complex essential for accurate splicing and also reveals that numerous cancer-associated mutations in both *SF3B1* and *SUGP1* all have the effect of disrupting this single protein–protein interaction, illustrating the critical nature of the SF3B1–SUGP1 interaction.

## Results

### The C-terminal region of SUGP1 is responsible for interaction with SF3B1

**Figure 1.**
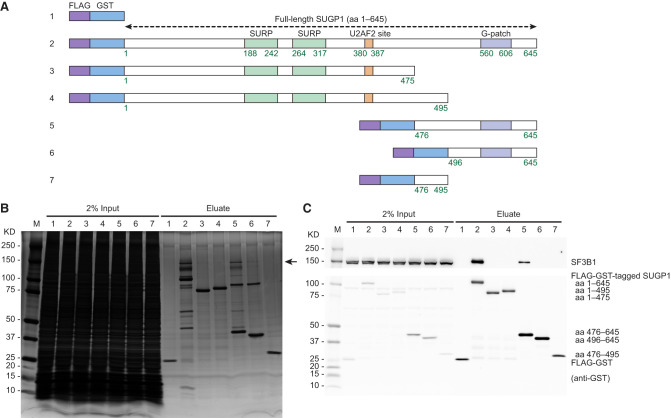
The C-terminal region of SUGP1 is responsible for interaction with SF3B1. (*A*) Schematic representation of plasmid constructs (1–7) encoding FLAG-GST tandem tags, FLAG-GST-tagged SUGP1 (full length), and its deletion mutants (amino acids 1–475, 1–495, 476–645, 496–645, and 476–495), respectively. (*B*,*C*) Each of the seven constructs was transfected into HEK293T cells, followed by affinity purification using FLAG and GST tags. Purified proteins were resolved by SDS-PAGE, followed by silver staining (*B*) or Western blotting (*C*). The black arrow in *B* points to the expected size of SF3B1. (M) Precision Plus protein marker (Bio-Rad).

An important prerequisite in understanding how *SF3B1* mutations that disrupt interaction with SUGP1 cause cancer is elucidating the molecular basis underlying how these two proteins interact with each other. To this end, we first set out to narrow down the region of SUGP1 that interacts with SF3B1 by using affinity purification of proteins associated with deletion variants of SUGP1. To do so, we made plasmid constructs encoding SUGP1 derivatives consisting of various truncations of SUGP1, each fused to tandem affinity tags FLAG (also known as DYKDDDDK) and glutathione S-transferase (GST) (Fig. 1A). We then transiently transfected each of these plasmids into HEK293T cells, harvested the cells, and used the whole-cell extracts to perform two rounds of affinity purification with anti-DYKDDDDK antibody and Glutathione Sepharose beads sequentially. By resolving the SUGP1-associated proteins by SDS-PAGE followed by silver staining or Western blotting, we found that SF3B1 copurified with full-length SUGP1 as expected (Fig. 1B,C). We also found that SF3B1 associates with the C-terminal region of SUGP1 (amino acids 476–645) but not with either of the N-terminal regions (amino acids 1–475 and 1–495) (Fig. 1B,C). A further truncated C-terminal fragment of SUGP1 (amino acids 496–645) did not copurify SF3B1 (Fig. 1B,C), suggesting that residues 476–495 are necessary for SF3B1 association. However, SUGP1 (amino acids 476–495) alone also did not copurify SF3B1 (Fig. 1B,C), suggesting that residues 476–495 are not sufficient for SF3B1 association and that sequences within the remaining part of SUGP1 (amino acids 476–645) are also required. This result prompted us to investigate the detailed molecular basis of the interaction between SF3B1 and the C-terminal region of SUGP1.

### Structural modeling independently identifies SUGP1 as a top SF3B1 interactor

**Figure 2.**
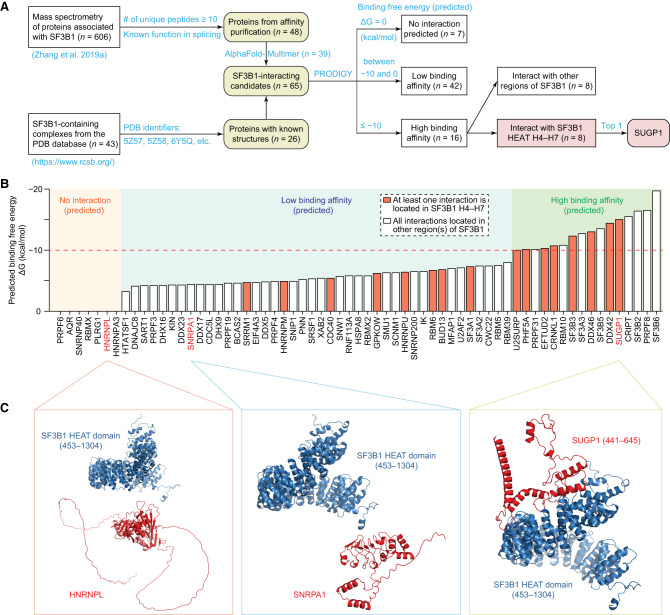
Structural modeling independently identified SUGP1 as a top SF3B1 interactor. (*A*) Computational workflow to identify SF3B1-interacting protein candidates. Potential SF3B1-interacting proteins (*n* = 65) were selected from the two sources as indicated. Protein complex structures were predicted using AlphaFold-Multimer, and the binding affinity of each complex was assessed using PRODIGY. (*B*) Predicted binding free energies of complexes by PRODIGY. SF3B1-interacting protein candidates were ranked by binding free energy and divided into three groups: no interaction (ΔG = 0 kcal/mol), low binding affinity (ΔG is between −10 and 0 kcal/mol), and high binding affinity (ΔG ≤ −10 kcal/mol). (*C*) Schematic drawings of the predicted structures of three example heterodimers in *B*. In all three examples, the SF3B1 HEAT domain (453–1304) is shown in blue, and its interacting protein candidate is shown in red. For clarity, SUGP1 (1–440), which does not participate in the SF3B1–SUGP1 interaction, is not shown in the *right* panel. However, the full-length SUGP1 is shown in Supplemental Figure S2A.

Given the difficulty of experimentally obtaining structures of complexes containing both SF3B1 and SUGP1 (as mentioned above), we decided to use computational modeling to gain structural insights into the SF3B1–SUGP1 contact interface to guide biochemical characterization of this critical interaction. To this end, we first set out to obtain a computationally derived baseline of binding free energy of SF3B1 with its interacting proteins. By manually searching all crystal/cryo-EM structures of complexes containing SF3B1 in the Protein Data Bank (PDB) (Berman et al. 2000), we obtained 43 complexes, from which we identified 26 proteins that have direct contacts with SF3B1 (Fig. 2A). As expected, SUGP1 was not in any of these complexes. To obtain other potential SF3B1-interacting proteins, we focused on proteins in affinity-purified spliceosomal complexes associated with SF3B1 (Zhang et al. 2019a). Among the 606 proteins identified by mass spectrometry that were associated with wild-type (WT) or K700E mutant SF3B1 (Zhang et al. 2019a), we selected protein candidates with sufficient abundance (at least 10 unique peptides recovered in the affinity purification–mass spectrometry) to minimize false positives and then manually excluded proteins that were never reported to be involved in splicing. We obtained a total of 48 candidate proteins (Fig. 2A), nine of which were also among the above-mentioned 26 proteins known to interact directly with SF3B1 in crystal/cryo-EM structures (e.g., other SF3B subunits: SF3B3, SF3B5, and PHF5A). The remaining 39 candidate proteins (including SUGP1) do not have crystal/cryo-EM structures to show their potential interactions with SF3B1. Therefore, we computationally modeled the structure of a potential heterodimeric complex for each of these candidate proteins with the SF3B1 HEAT domain (amino aicds 453–1304) using AlphaFold-Multimer (Evans et al. 2022). We chose AlphaFold-Multimer because it has remarkably high accuracies for structural predictions (see above). Furthermore, our own evaluation of this method on three known heterodimers (without using their known structures for training or as templates for predictions) also showed that the predicted structures have a high accuracy of only ∼0.4–1.2 Å Cα root-mean-square deviation (RMSD) when compared with the known structures (Supplemental Fig. S1A–C). These high-accuracy results suggest that the SF3B1 complex structures predicted by AlphaFold-Multimer are worthy of further investigation.

To evaluate the protein–protein interactions in the above-mentioned 65 SF3B1-containing heterodimer complexes (26 with known structures and 39 with predicted structures), we predicted the binding free energy for each of them using PRODIGY (Xue et al. 2016). The results are shown in Figure 2B and Supplemental Table S1. We found that 16 proteins interact with SF3B1 with predicted high binding affinity (Fig. 2B,C), eight of which have at least one direct interaction with SF3B1 HEAT repeats H4–H7, the hotspot area for cancer mutations (Cretu et al. 2016). Significantly, SUGP1 was identified as the top interactor among proteins with direct interactions with repeats H4–H7 (Fig. 2B,C; Supplemental Fig. S2A). By comparing subportions of the modeled SF3B1–SUGP1 heterodimer with the cryo-EM structure of SF3B1 in the PDB (ID 6AHD) (Zhan et al. 2018), we found that the predicted structure of SF3B1 HEAT repeats H4–H7 aligns extremely well with the known structure (0.64 Å RMSD) (Supplemental Fig. S2B). This was also the case for SF3B1 HEAT domain residues 590–1304 (a continuous region without missing residues in the cryo-EM structure), with only 1.7 Å RMSD (Supplemental Fig. S2C). We reasoned that this modeled SF3B1–SUGP1 heterodimeric structure is ideal for experimental validation.

### Two separate regions of SUGP1 flanking the G-patch directly interact with SF3B1

**Figure 3.**
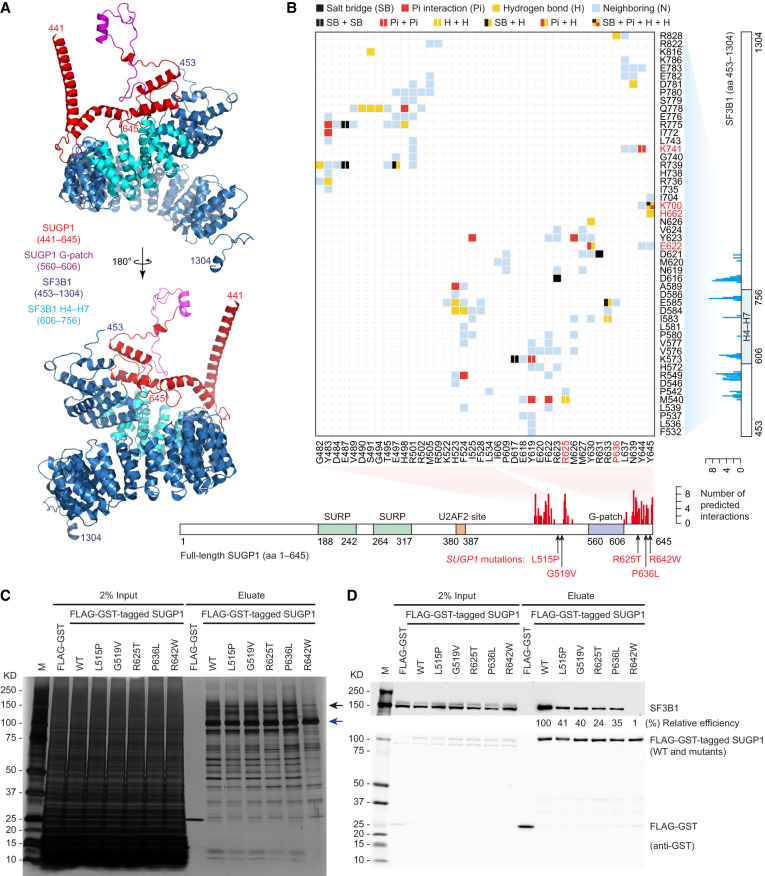
Two separate regions of SUGP1 flanking the G-patch directly interact with SF3B1. (*A*) Schematic drawing of the predicted structure of the SF3B1–SUGP1 heterodimer. The SF3B1 HEAT domain (453–1304) is shown in blue, with SF3B1 H4–H7 (606–756) shown in cyan. SUGP1 (441–645) is indicated in red, with the G-patch (560–606) shown in purple. For clarity, SUGP1 (1–440), which does not participate in the interaction, is not shown. (*B*) Matrix of predicted interactions in the SF3B1–SUGP1 interface, analyzed by Discovery Studio (BIOVIA). Each row represents one residue of SF3B1 (with hotspot residues in HEAT repeats H4–H7 marked in red), and each column represents one residue of SUGP1 (with cancer-associated residues marked in red). Total numbers of predicted interactions on SF3B1 residues are shown in a cyan bar graph (*right*), and those on SUGP1 residues are shown in a red bar graph (*bottom*). The five cancer-associated mutations in *SUGP1* are shown in red. (*C*,*D*) HEK293T cells were transfected with expression plasmid for FLAG-GST tandem tags or FLAG-GST-tagged SUGP1 (WT or one of its mutants as indicated), followed by affinity purification using FLAG and GST tags. Purified proteins were resolved by SDS-PAGE, followed by silver staining (*C*) or Western blotting (*D*). The black arrow in *C* points to the expected size of SF3B1, and the blue arrow points to the expected size of FLAG-GST-tagged SUGP1. (*D*) The relative efficiency of copurification of SF3B1 with each SUGP1 variant relative to WT (mean of two independent experiments) is shown *below* the SF3B1 blot.

A powerful approach to validate the predicted interactions in the modeled SF3B1–SUGP1 complex is to substitute and/or delete the interacting residues and determine whether the interaction is disrupted. To this end, we first identified the interacting residues by analyzing the predicted interface between SF3B1 and SUGP1 in the modeled heterodimer and found that two separate regions of SUGP1 (amino acids 482–534 and 606–645) have extensive interactions with SF3B1 (Fig. 3A,B). Importantly, both of these regions are located in the C-terminal quarter of SUGP1, which is highly consistent with our experimental data showing that SUGP1 (amino acids 476–645) is responsible for SF3B1 association in our deletion analyses above.

Strikingly, we found that these two SF3B1-interacting regions are located both upstream of and downstream from the SUGP1 G-patch but not within it (Fig. 3A,B). In our previous study, we identified five naturally occurring *SUGP1* missense mutations (L515P, G519V, R625T, P636L, and R642W) flanking the G-patch in cancers that partially recapitulate mutant SF3B1 splicing dysregulation (Liu et al. 2020). Remarkably, all five of these *SUGP1* mutations are located in the two SF3B1-interacting regions, and we thus wanted to investigate whether these cancer-associated mutations disrupt the interaction of SUGP1 with SF3B1. To test this, we made plasmid constructs encoding the five SUGP1 mutants, each fused to tandem affinity tags (FLAG and GST), and then performed two rounds of affinity purification using the same method as described above. We found that all five *SUGP1* mutations weakened interaction with SF3B1 (Fig. 3C,D). Notably, the R642W mutation, which is located near the very C terminus of the 645-residue protein, almost completely disrupted the interaction (Fig. 3C,D). This result suggests that the cancer-associated *SUGP1* mutations in the two SF3B1-interacting regions function by weakening the SUGP1 interaction with SF3B1 rather than by affecting the function of the G-patch, as we had previously suggested (Liu et al. 2020).

### SF3B1 hotspot residues interact with the very C terminus of SUGP1

**Figure 4.**
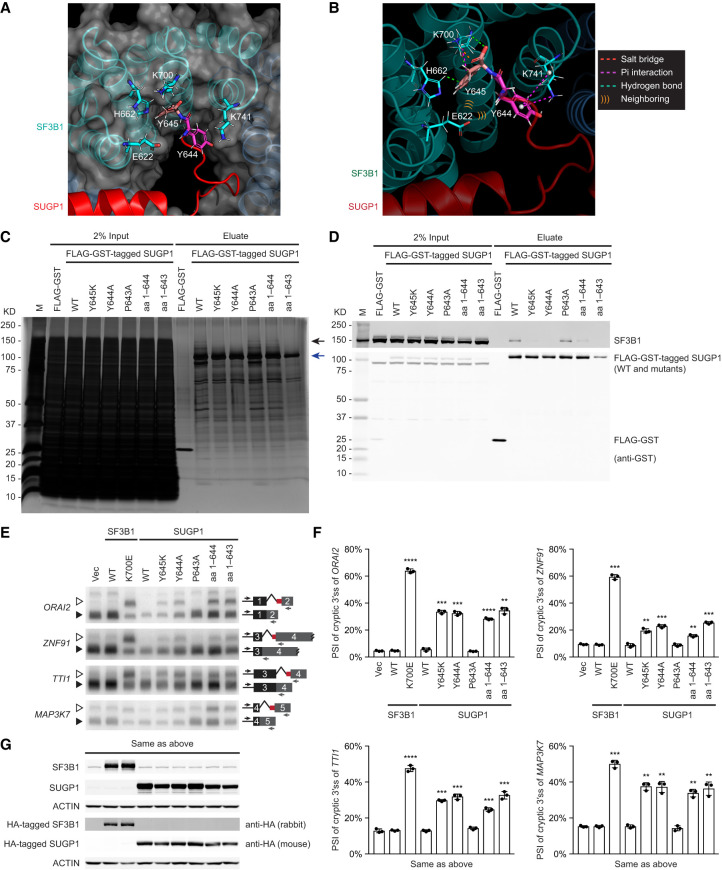
SF3B1 hotspot residues interact with the very C terminus of SUGP1. (*A*) AlphaFold-Multimer-predicted interface between the C terminus of SUGP1 and the SF3B1 hotspot region. SF3B1 is shown in cyan, and SUGP1 is shown in red. The surface of SF3B1 is shown as a gray semitransparent shell. (*B*) AlphaFold-Multimer-predicted interactions between the C-terminal residues of SUGP1 and SF3B1 hotspot residues. (*C*,*D*) HEK293T cells were transfected with expression plasmid for FLAG-GST tandem tags or FLAG-GST-tagged SUGP1 (WT or one of its mutants, as indicated), followed by affinity purification using FLAG and GST tags (but with 300 mM NaCl instead of 150 mM). Purified proteins were resolved by SDS-PAGE, followed by silver staining (*C*) or Western blotting (*D*). The black arrow in *C* points to the expected size of SF3B1, and the blue arrow points to the expected size of FLAG-GST-tagged SUGP1. (*E*) Empty vector plasmid (Vec) or expression plasmid for HA-tagged SF3B1 (WT or K700E) or HA-tagged SUGP1 (WT or one of the mutants, as indicated) was cotransfected with a mixture of four minigenes (*ORAI2*, *ZNF91*, *TTI1*, and *MAP3K7*) in HEK293T cells, followed by ^32^P RT-PCR to detect cryptic (open arrowheads) and canonical (solid arrowheads) 3′ splice sites of the minigene transcripts, as indicated. (*F*) Quantification of the ^32^P RT-PCR products in *E*. Percent spliced in (PSI) of the cryptic 3′ splice sites of the indicated minigenes is shown. Bars represent the mean ± SD (*n* = 3; three independent experiments). Unpaired, two-tailed, and unequal variance *t*-tests were performed to determine the statistical difference between Vec and each of the expression plasmids. (**) *P* < 0.01, (***) *P* < 0.001, (****) *P* < 0.0001. (*G*) Western blotting of protein extracts from HEK293T cells in *E*. The SF3B1 and SUGP1 blots show both endogenous and exogenous proteins, whereas the anti-HA (rabbit) and anti-HA (mouse) blots show only the exogenously expressed HA-tagged proteins.

Based on the predicted SF3B1–SUGP1 heterodimer structure, the two SF3B1-interacting regions are extensive, not only including residues near the G-patch but also extending further upstream to SUGP1 Gly482 and downstream to the C terminus (Fig. 3A,B). Surprisingly, the SUGP1 C terminus is located near a “pocket” encompassing the *SF3B1* hotspot mutations (Fig. 4A). Specifically, the last residue (Y645) of SUGP1 is predicted to make multiple contacts (including one salt bridge interaction, one Pi interaction, and two hydrogen bonds) with SF3B1 K700 and also to form a hydrogen bond with hotspot residue SF3B1 H662 (Fig. 4B). The second to last SUGP1 residue (Y644) makes two Pi interactions with hotspot residue SF3B1 K741, and both SUGP1 Y645 and Y644 are located closer than 6 Å (“neighboring”) to hotspot residue SF3B1 E622 (Fig. 4B).

To validate the critical role of these two highly conserved C-terminal SUGP1 residues, we mutated each of them individually or deleted one or both to examine whether the interaction of SUGP1 with SF3B1 would be disrupted. Specifically, we made plasmid constructs that encoded tandem affinity tags (FLAG and GST) fused to each of the different SUGP1 mutant derivatives. These included missense mutants of the last residue (Y645K), the second to last residue (Y644A), and the third to last residue (P643A; note that P643 is not predicted to interact with SF3B1), as well as deletion of the last residue (amino acids 1–644) and deletion of the last two residues (amino acids 1–643). By performing two rounds of affinity purification using the same method as described above (except with 300 mM NaCl instead of 150 mM), we found that both the Y645K and Y644A mutations disrupted the interaction of SUGP1 with SF3B1, whereas P643A did not (Fig. 4C,D). Likewise, deletion of either the last residue or the last two residues disrupted the interaction (Fig. 4C,D).

We next wished to investigate whether these SUGP1 mutants recapitulate the splicing defects (i.e., cryptic 3′ss usage) of K700E mutant SF3B1. To this end, we cotransfected each of the plasmids expressing the above-mentioned SUGP1 mutants (as well as WT and K700E mutant SF3B1) into HEK293T cells together with minigenes of four of the mutant SF3B1 target transcripts that we used in our previous study (Zhang et al. 2019a). We then purified RNAs from the transfected cells and performed ^32^P RT-PCR to detect cryptic 3′ss usage in pre-mRNAs produced from the minigenes. Consistent with the results of our previous study (Zhang et al. 2019a), K700E mutant SF3B1 induced robust use of cryptic 3′ splice sites in all the minigenes (Fig. 4E–G). Significantly, both the missense mutants (Y645K and Y644A) and the deletion mutants (amino acids 1–644 and 1–643) of SUGP1 induced use of the cryptic 3′ splice sites, whereas the P643A mutant did not (Fig. 4E–G).

Together, the above results not only confirmed that the very C-terminal Tyr residues of SUGP1 are necessary for both SUGP1 interaction with SF3B1 and accurate splicing but also provided strong support for the accuracy of the predicted SF3B1–SUGP1 heterodimer structure.

### Characterization of the region upstream of the SUGP1 G-patch for SF3B1 interaction

**Figure 5.**
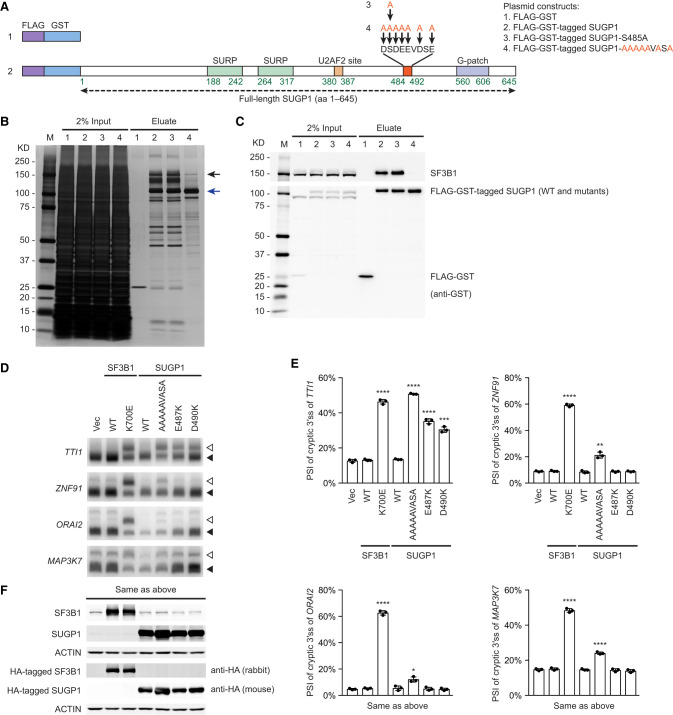
Characterization of the region upstream of the SUGP1 G-patch for SF3B1 interaction. (*A*) Schematic representation of plasmid constructs (1–4) encoding FLAG-GST tandem tags, FLAG-GST-tagged SUGP1, and its mutants (S485A and AAAAAVASA), respectively. (*B*,*C*) Each of the four constructs was transfected into HEK293T cells, followed by affinity purification using FLAG and GST tags. Purified proteins were resolved by SDS-PAGE, followed by silver staining (*B*) or Western blotting (*C*). The black arrow in *B* points to the expected size of SF3B1, and the blue arrow points to the expected size of FLAG-GST-tagged SUGP1. (*D*) Vec or expression plasmid for HA-tagged SF3B1 (WT or K700E) or HA-tagged SUGP1 (WT or one of the mutants as indicated) was cotransfected with a mixture of four minigenes (*TTI1*, *ZNF91*, *ORAI2*, and *MAP3K7*) in HEK293T cells, followed by ^32^P RT-PCR to detect cryptic (open arrowheads) and canonical (solid arrowheads) 3′ splice sites of the minigene transcripts as indicated. (*E*) Quantification of the ^32^P RT-PCR products in *D*. PSI of the cryptic 3′ splice sites of the indicated minigenes is shown. Bars represent the mean ± SD (*n* = 3; three independent experiments). Unpaired, two-tailed, and unequal variance *t*-tests were performed to determine the statistical difference between Vec and each of the expression plasmids. (*) *P* < 0.05, (**) *P* < 0.01, (***) *P* < 0.001, (****) *P* < 0.0001. (*F*) Western blotting of protein extracts from HEK293T cells in *D*. The SF3B1 and SUGP1 blots show both endogenous and exogenous proteins, whereas the anti-HA (rabbit) and anti-HA (mouse) blots show only the exogenously expressed HA-tagged proteins.

After characterizing interactions involving the region downstream from the SUGP1 G-patch, we next wanted to validate critical interactions in the upstream region. Our deletion analyses above showed that residues 476–495 of SUGP1 are necessary for SF3B1 association (Fig. 1B,C). Consistent with that result, the predicted SF3B1–SUGP1 interface extends further upstream to SUGP1 Gly482 (Fig. 3A,B). We manually examined this region and found a highly negatively charged amino acid sequence, DSDEEVDSE (amino acids 484–492), where Ser485 is phosphorylated and therefore also negatively charged (UniProt Consortium 2023). To test whether this sequence is important for SF3B1 interaction, we first made SUGP1 mutant plasmid constructs (fused with FLAG and GST affinity tags) that changed either all of the negatively charged residues of this sequence to alanine (i.e., AAAAAVASA) or only Ser485 to alanine (S485A) (Fig. 5A) and then performed two rounds of affinity purification using the standard method above. We found that the AAAAAVASA mutant SUGP1 almost completely lost interaction with SF3B1, whereas S485A did not (Fig. 5B,C). To examine the contribution of each of these negatively charged residues, we made mutant plasmids that each mutated only one of the seven negatively charged residues and performed two rounds of affinity purification as above. We found that only E487K and D490K noticeably weakened the interaction of SUGP1 with SF3B1 (Supplemental Fig. S3A,B). This result is consistent with the modeled SF3B1–SUGP1 heterodimer structure, which shows that SUGP1 E487 has strong bonding with both Arg775 (a hotspot residue) and Arg739 of SF3B1 (each with two salt bridge interactions) and that SUGP1 D490 forms a hydrogen bond with SF3B1 Gln778 (Fig. 3B).

We next asked whether the above-mentioned *SUGP1* mutations that disrupted SUGP1–SF3B1 interaction could also recapitulate the splicing defects of mutant SF3B1. To this end, we cotransfected each of the three SUGP1 mutants (AAAAAVASA, E487K, and D490K) into HEK293T cells together with the four minigenes harboring mutant SF3B1 target cryptic 3′ splice sites. We then analyzed splicing of the minigenes by ^32^P RT-PCR as above. Importantly, we found that all three SUGP1 mutants partially recapitulated the splicing defects of mutant SF3B1 (e.g., in the *TTI1* minigene) (Fig. 5D–F).

### The SF3B1–SUGP1 interaction facilitates formation of a larger complex required for accurate splicing

**Figure 6.**
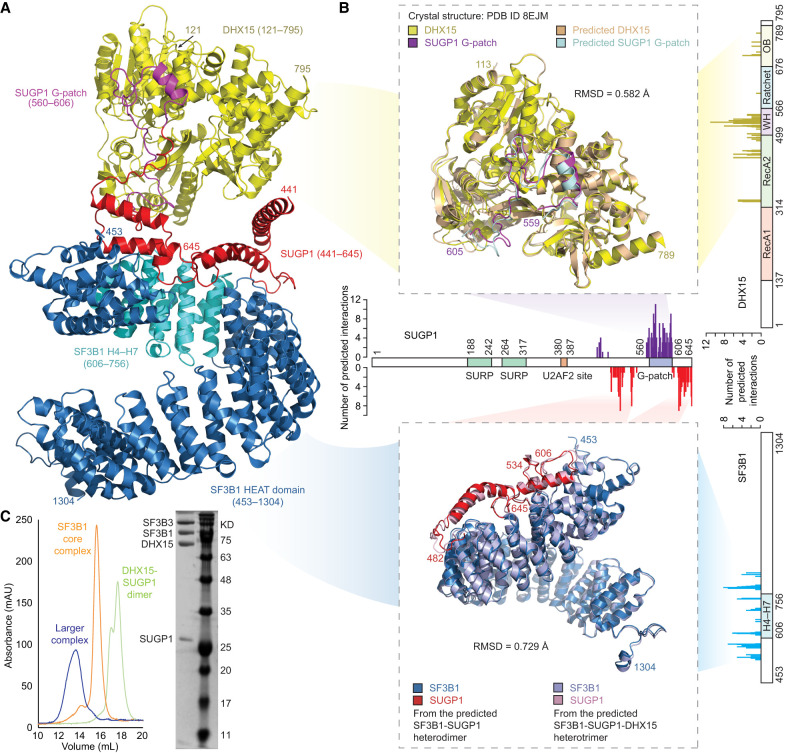
The SF3B1–SUGP1 interaction facilitates formation of a larger complex required for accurate splicing. (*A*) Schematic drawing of the predicted structure of the SF3B1–SUGP1–DHX15 heterotrimer. The SF3B1 HEAT domain (453–1304) is shown in blue, with SF3B1 H4–H7 (606–756) shown in cyan. SUGP1 (441–645) is indicated in red, with the G-patch (560–606) shown in purple. DHX15 (121–795) is yellow. For clarity, SUGP1 (1–440) and DHX15 (1–120), which do not participate in these interactions, are not shown. (*B*, *top*) Similarities between the predicted structure and the crystal structure of the DHX15–SUGP1 G-patch complex. Note that only the residues present in the crystal structure (PDB ID 8EJM)—i.e., DHX15 (113–789) and SUGP1 (559–605)—are shown in the structure alignment. (*Bottom*) Similarities of the SF3B1–SUGP1 interface between the predicted heterodimer and predicted heterotrimer complexes, as indicated. The two separate SF3B1-interacting regions in SUGP1 (482–534 and 606–645) are shown. Bar graphs represent the total numbers of predicted interactions on residues of the SF3B1 HEAT domain (*bottom right*), SUGP1 (*middle*), and DHX15 (*top right*). (WH) Winged helix, (OB) oligonucleotide/oligosaccharide binding. (*C*, *left*) Gel filtration profile of the complex of SF3B together with DHX15–SUGP1 (blue), the SF3B core complex alone (orange), and the DHX15–SUGP1 heterodimer alone (green). (*Right*) Fractions containing the complex of SF3B together with DHX15–SUGP1 were pooled, concentrated, and rerun on the Superose 6 column. Peak fractions with high absorbance at 280 nm were concentrated and run on a 12% SDS-PAGE gel. BLUEstain protein ladder (Goldbio) was used. (SF3B3) SF3B3 (1–1217), (SF3B1) SF3B1 HEAT domain (453–1304), (DHX15) DHX15 (113–795), (SUGP1) SUGP1 (433–645).

Given that SF3B1 functions in the context of the complex spliceosome, we next wanted to know whether the modeled SF3B1–SUGP1 heterodimer structure is compatible with formation of a larger complex required for RNA splicing; i.e., accurate 3′ss selection. Specifically, our recent study showed that the RNA helicase DHX15 is involved in SUGP1-mediated RNA splicing by binding to the SUGP1 G-patch (Zhang et al. 2022a). Our modeled SF3B1–SUGP1 heterodimer structure showed that the two SF3B1-interacting regions in SUGP1 closely surround, but do not overlap, the G-patch (Fig. 3A,B). We therefore asked whether DHX15 is able to fit into this structure. By again using AlphaFold-Multimer, we were able to successfully model the structure of the SF3B1–SUGP1–DHX15 trimer (Fig. 6A; Supplemental Movie S1). Strikingly, as shown in Figure 6B, the predicted DHX15–SUGP1 G-patch interface in this trimeric structure aligns nearly perfectly (0.58 Å RMSD) with that in the crystal structure of the DHX15–SUGP1 G-patch complex (PDB ID 8EJM) that we experimentally determined in our recent study (Zhang et al. 2022a). Concurrently, the SF3B1–SUGP1 interface in the predicted trimer remains almost the same (0.73 Å RMSD) as in the predicted SF3B1–SUGP1 heterodimer that we biochemically characterized above (Fig. 6B). These findings suggest not only that the SF3B1–SUGP1 heterodimer structure that we modeled (and experimentally validated) is indeed compatible with DHX15 binding to form a larger complex but also that the binding of SF3B1 to SUGP1 in two separate regions (both subject to multiple cancer-associated mutations) functions to “loop out” the SUGP1 G-patch for interaction with DHX15.

To examine experimentally whether SF3B1, SUGP1, and DHX15 can indeed form a larger complex, we first coexpressed DHX15 (amino acids 113–795), which lacks the N-terminal unstructured region (Murakami et al. 2017; Studer et al. 2020), with SUGP1 (amino acids 433–645, which include the G-patch) in insect cells and then purified the DHX15–SUGP1 heterodimer as described (Zhang et al. 2022a). We also purified the SF3B core complex (Cretu et al. 2016) by coexpressing the C-terminal HEAT domain of SF3B1 (amino acids 453–1304), SF3B3 (amino acids 1–1217), SF3B5 (amino acids 1–86), and PHF5A (amino acids 1–98) in insect cells, followed by purification as described in the Materials and Methods. By mixing the purified SF3B core complex with the purified DHX15–SUGP1 heterodimer, we found that a larger complex (containing at least SUGP1, DHX15, the SF3B1 HEAT domain, and SF3B3) was formed, based on gel filtration chromatography (Fig. 6C). Because a main function of SF3B3 is to interact with both ends of the SF3B1 HEAT domain to stabilize its superhelical structure (Cretu et al. 2016), it is likely that the formation of the larger complex is solely mediated by the direct interaction between SUGP1 and SF3B1 that we have characterized.

## Discussion

We have used structural modeling with AlphaFold-Multimer and experimental analyses to characterize the SF3B1–SUGP1 interaction. Our study has provided a remarkable molecular and mechanistic understanding of this critical interaction and of how numerous mutations in both proteins disrupt it and thereby cause cancer-relevant RNA missplicing. Two separate regions of SUGP1, including two tyrosines at the very C terminus, are essential for interaction with the region of SF3B1 that harbors cancer hotspot mutations. Moreover, the direct residue–residue contacts between SF3B1 and SUGP1 all flank the SUGP1 G-patch but are not within it, allowing the SUGP1 G-patch to “loop out” to interact with DHX15. The predicted SF3B1–SUGP1–DHX15 trimer forms a sandwich-like structure with SUGP1 in the middle, such that SUGP1 forms a bridge to couple SF3B1 with DHX15 during branch site recognition in the early stage of spliceosome assembly. The loss of interaction between SF3B1 and SUGP1 due to cancer mutations in either *SF3B1* or *SUGP1* provides an explanation for why the mutant spliceosome fails to activate DHX15 for ATP hydrolysis required for canonical branch site selection.

To our knowledge, our study is the first to use AlphaFold-Multimer modeling to accurately predict the structure of a splicing-related multiprotein complex. Given that this reflects a new approach to structure prediction, it is important to validate its accuracy, which we did in several ways. First, subportions of the predicted complexes align well with the experimentally determined cryo-EM/crystal structures. Second, we experimentally validated the critical interactions of specific SUGP1 residues with residues in SF3B1, as substitutions or deletions of these residues all disrupted SUGP1 interaction with SF3B1. Third, substitutions/deletions of the critical residues in SUGP1, including naturally occurring cancer mutations, recapitulated the RNA missplicing induced by *SF3B1* cancer mutations that were previously shown to disrupt SF3B1 interaction with SUGP1 (Zhang et al. 2019a). Our success thus encourages an alternative way of studying interactions, including transient ones, within spliceosomal complexes and indeed other multisubunit complexes whose structures are challenging to capture experimentally.

We have shown that numerous *SUGP1* mutations affecting residues critical for binding SF3B1 recapitulate the splicing defects of hotspot *SF3B1* cancer mutations, confirming the cooperative role of these two proteins in 3′ss selection. However, this finding raises somewhat of a conundrum: If these mutations disrupt interaction with SF3B1, and hence SUGP1 mutants do not associate with the spliceosome, how, then, do they affect splicing? Unlike SUGP1 knockdown cells, which lack the protein, the mutant SUGP1 transfected cells maintain essentially WT levels of endogenous SUGP1, which should continue to provide function if the mutant proteins are simply inactive. A likely explanation stems from the fact that SUGP1 has a binding site for U2AF2 (U2 small nuclear RNA auxiliary factor 2) and two SURP domains (Sampson and Hewitt 2003; Zhang et al. 2019a). Because SURP domains are known to interact with the branch site-binding protein splicing factor 1 (SF1) (Crisci et al. 2015), the SUGP1 mutants would still localize to the branch site via interactions with SF1 and U2AF2 at the earliest stage in branch site recognition and in the absence of interaction with SF3B1. The SUGP1 mutants would then be defective in stably recruiting SF3B1-containing U2 snRNP, thereby preventing—or at least slowing—utilization of the canonical branch site. U2 snRNP will then recognize an unblocked alternative branch site, if available, and the spliceosome will ultimately use a cryptic 3′ss—again, if present. This model not only explains the apparent paradox suggested by our data but also suggests that SUGP1, together with DHX15 (Zhang et al. 2022a), functions at the earliest stage of branch site selection, as we discuss further below.

Our data showing that the SF3B1 binding-defective SUGP1 mutants nonetheless affect splicing are difficult to reconcile with models suggesting that SUGP1 functions later in splicing; i.e., in quality control (QC) by activating DHX15-dependent dissociation of defectively assembled spliceosomes (Beusch et al. 2023; Feng et al. 2023). Such a model would require direct interaction of SUGP1 with U2 snRNP/SF3B1, which the mutants cannot do. Additionally, as levels of endogenous SUGP1 are essentially unchanged in the mutant-expressing cells, the WT protein should still be able to function in QC, thereby preventing the cryptic splicing that we observed in these cells. Thus, we believe that SUGP1–DHX15 functions during the earliest step in branch site recognition rather than later in QC. Besides, no purified 17S U2 snRNPs have been found to contain SUGP1 (Zhang et al. 2020, 2022b; Tholen et al. 2022), consistent with the idea that SUGP1 functions in an earlier step in splicing. However, we do not rule out the possibility that DHX15 (although not SUGP1) may be involved in QC at a later step. This possibility is consistent with suggestions from previous studies (Fourmann et al. 2016; Maul-Newby et al. 2022), with our findings that DHX15 associates with spliceosomes independently of SUGP1 (Zhang et al. 2019a, 2022a), and with the fact that DHX15 can interact with multiple other G-patch-containing proteins (Hegele et al. 2012).

Our study provides important insights into a critical early step in branch site recognition, which is the “handover” of the branch site from SF1 to the branchpoint-interacting stem–loop (BSL) of U2 snRNA. It is believed that at least two steps are involved in this process, with the first step bringing U2 snRNP to the vicinity of the branch site and the second step opening up the BSL for base-pairing with the branch site (Zhang et al. 2020, 2022b; Tholen et al. 2022). For the second step, recent studies of U2 snRNP structures suggested that the RNA helicase DDX46 (also known as PRP5) likely unwinds the double-stranded BSL and consequently displaces the BSL-interacting protein HTATSF1 (Zhang et al. 2020, 2022b; Tholen et al. 2022). For the first step, it was suggested that one or more of several SURP domain-containing proteins localize U2 snRNP to the vicinity of the branch site by binding to SF1 (Crisci et al. 2015). Our results strongly suggest that SUGP1 is the SURP-containing protein that brings U2 snRNP to the canonical branch site, for the reasons mentioned above. All of the U2 snRNPs with known structures are of late stage and contain DDX46 but not SUGP1 (Zhang et al. 2020, 2022b; Tholen et al. 2022), suggesting that SUGP1 is discharged before DDX46 is recruited to U2 snRNP. In fact, DDX46 has an acidic loop region that binds SF3B1 also in the hotspot area (including residue K700) (Zhang et al. 2022b), and therefore the recruitment of DDX46 will only be possible after dissociation of SUGP1 from the SF3B1 hotspot region.

How, then, does SUGP1–DHX15 function in branch site recognition? We envision a two-step scenario in which in the first step SUGP1 recruits U2 snRNP to the branch site and then activates DHX15, which “pulls” the pre-mRNA into the RNA path of SF3B1 until the branch site is close to the BSL. Following dissociation of SUGP1 from the complex, DDX46 is recruited to U2 snRNP, likely by its direct interaction with SF3B1 (Zhang et al. 2022b), where it opens up the BSL to allow formation of the U2 snRNA/branch site duplex. In this way, SUGP1–DHX15 works on the pre-mRNA during the first step of branch site recognition, and DDX46 rearranges U2 snRNA during the second step. It is the first step that mainly determines the selection of correct branch sites, consistent with the fact that all *SUGP1* cancer-associated mutations (as well as additional mutations described in this study) partially recapitulate the mutant SF3B1 missplicing pattern (Liu et al. 2020; Alsafadi et al. 2021). Furthermore, SUGP1 knockdown strongly phenocopies mutant SF3B1 (Zhang et al. 2019a; Alsafadi et al. 2021), including robust usage of cryptic 3′ splice sites in all of the top mutant SF3B1 targets tested (Zhang et al. 2019a). While it has been suggested that loss of DDX46 may affect some aberrantly spliced transcripts found in hematological malignancies (Zhang et al. 2022b), knockdown of DDX46, unlike of SUGP1, did not induce cryptic 3′ss usage in any of the aforementioned top targets of mutant SF3B1 (Supplemental Fig. S4). Additionally, unlike both *SUGP1* and *DHX15* (Liu et al. 2020; Alsafadi et al. 2021; Zhang et al. 2022a), *DDX46* is not known to harbor any cancer-associated mutations that recapitulate mutant SF3B1 missplicing.

It is remarkable that numerous different cancer-associated mutations in *SUGP1* as well as in *SF3B1* function by the same mechanism; i.e., by disrupting or weakening the interaction between the two proteins. Previous analysis of *SF3B1* cancer-associated mutations showed that the majority of the >40 mutated residues are located in SF3B1 HEAT repeats H4–H7 (Cretu et al. 2016). Some of these SF3B1 hotspot residues (e.g., K700, H662, and K741) were predicted by AlphaFold-Multimer to have direct contacts with SUGP1, while others were not. We assume that at least some of these other mutations (e.g., E622D, R625C, and K666N, which we showed previously disrupt interaction with SUGP1) (Zhang et al. 2019a) may perturb the local structure of SF3B1 H4–H7 and thereby dislodge SUGP1 due to the proximities of these residues to the mutational “pocket.” For example, SF3B1 E622 is predicted to be close (“neighboring”) to Y644 and Y645 of SUGP1. Likewise, among the five *SUGP1* cancer-associated residues, only R625 and P636 were predicted to have direct interactions with SF3B1, while the other three (L515, G519, and R642) were not (although embedded in the interface). Cancer mutations at these three residues may also disrupt the SUGP1–SF3B1 interaction by perturbing the local structure of the interface. For example, R642 is located extremely close to the very C terminus of SUGP1, and therefore its mutation (R642W) may alter the positions of the two C-terminal tyrosines. Last, hotspot mutations located in other SF3B1 HEAT repeats (e.g., E592K and E902K) are likely to disrupt splicing by different mechanisms (Seiler et al. 2018; Choi et al. 2023). An important goal for future studies will be to elucidate these specific mechanisms. This will be valuable not only from the cancer perspective but also because of the insights they will likely provide into the earliest steps in splicing, similar to our studies on the SF3B1–SUGP1 interaction.

In summary, we have characterized a multiprotein complex essential for accurate 3′ss selection. Our work exemplifies the use of AlphaFold-Multimer to accurately model multisubunit protein complexes, provides important new insights into how the complex mediating accurate branch site selection assembles and functions, and has shown that numerous cancer-associated mutations in both *SF3B1* and *SUGP1* misregulate RNA splicing by disrupting a single protein–protein interaction.

## Materials and methods

### Cell lines

HEK293T cells were cultured in Dulbecco's modified Eagle's medium supplemented with 10% fetal bovine serum at 37°C in a 5% CO_2_ incubator.

### Computational identification of SF3B1-interacting protein candidates and structural modeling of protein complexes

SF3B1-interacting protein candidates were selected from the following two sources: proteins shown to directly interact with SF3B1 in crystal/cryo-EM structures of multiprotein complexes in the PDB (Berman et al. 2000) and proteins associated with SF3B1 identified by affinity purification–mass spectrometry (Zhang et al. 2019a). From the former source, we obtained heterodimeric structures of SF3B1 and each of its interacting proteins (*n* = 26) by extracting their atomic coordinates directly from the structures of the larger complexes deposited in the PDB (in cases where the SF3B1-interacting proteins [e.g., SF3B core proteins] are present in multiple complexes, we extracted their atomic coordinates from a representative structure). From the latter source, we first selected proteins with at least 10 unique peptides recovered in affinity purification–mass spectrometry and then manually excluded those without a known function in splicing based on the functional annotation by UniProt (https://www.uniprot.org). Next, structural modeling of heterodimer complexes between the SF3B1 HEAT domain (amino acids 453–1304) and each of these SF3B1-interacting protein candidates (*n* = 39) was performed using AlphaFold-Multimer v2.1.0 (Evans et al. 2022) with default settings (as well as the needed databases specific to this version), except that the -t (max_template_date) parameter was set to 2021-11-01 and the -m (model_preset) parameter was set to multimer. For each complex, AlphaFold-Multimer generated 25 predicted structures, from which the best one by ranking was selected for downstream analyses. Finally, binding free energies of the above-mentioned 65 SF3B1-containing heterodimer complexes (i.e., 26 extracted from the PDB and 39 predicted by AlphaFold-Multimer) were predicted using PRODIGY (Xue et al. 2016) with the temperature set to 25°C. Structural modeling of the three known heterodimer complexes in Supplemental Figure S1, as well as the heterotrimer complex of SF3B1 HEAT domain–SUGP1–DHX15, was performed using AlphaFold-Multimer (Evans et al. 2022), with the same parameters as above.

### Structure alignment and RMSD calculations

Structure alignment of protein complexes and RMSD calculations were performed using PyMOL (http://www.pymol.org/pymol) with the following parameters: cutoff = 2.0, cycles = 5, matrix = “BLOSUM62,” quiet = 0, and transform = 1. In cases where the crystal/cryo-EM structures have missing residues, the corresponding unpaired residues in the predicted structures were removed prior to alignment.

### Protein interface analysis and visualization

Interactions in protein–protein interfaces were analyzed using the Discovery Studio 2018 software package (BIOVIA). The following parameters were used: maximum hydrogen bond distance = 3.4, maximum weak hydrogen bond distance = 3.8, maximum salt bridge distance = 4.0, interface definition = “contact area,” surface area probe radius = 0.6, and percent contact area threshold = 0. Visualization of protein–protein interactions was performed using PyMOL (http://www.pymol.org/pymol).

### Expression plasmid constructs

HA-tagged SF3B1 WT and mutant K700E, HA-tagged SUGP1 WT and mutants (L515P, G519V, R625T, P636L, and R642W), and FLAG-GST tandem tags were cloned in our previous studies (Zhang et al. 2019a, 2022a; Liu et al. 2020). FLAG-GST-tagged SUGP1 and its missense mutants (P643A, Y644A, and Y645K) and deletion mutants (amino acids 1–475, 1–495, 476–645, 496–645, 476–495, 1–643, and 1–644), as well as HA-tagged SUGP1 missense mutants (P643A, Y644A, and Y645K) and deletion mutants (amino acids 1–643 and 1–644), were cloned in p3xFLAG-CMV-14 (Sigma-Aldrich). FLAG-GST-tagged SUGP1 mutants (S485A, S485K, and AAAAAVASA) and HA-tagged SUGP1 mutants (D484K, D486K, E487K, E488K, D490K, and E492K) were generated by overlap extension PCR (Ho et al. 1989). FLAG-GST-tagged SUGP1 missense mutants (L515P, G519V, R625T, P636L, R642W, D484K, D486K, E487K, E488K, D490K, and E492K) were generated by replacing the WT fragment between the EcoRI and BamHI sites with the mutant fragment double-digested from each of the corresponding HA-tagged SUGP1 mutant constructs. HA-tagged SUGP1 mutant AAAAAVASA was generated by replacing the WT fragment between the EcoRI and BamHI sites with the mutant fragment double-digested from the FLAG-GST-tagged SUGP1 mutant AAAAAVASA. In all the constructs above, tags were added to the N terminus, and two stop codons were added immediately following the last codon of each protein.

### Western blotting

Western blotting was performed as described (Zhang et al. 2019a) with the following primary antibodies: anti-SF3B1 (1:1000; Bethyl Laboratories A300-996A), anti-GST (1:1500; Invitrogen A5800), anti-SUGP1 (1:1000; Bethyl Laboratories A304-675A-M), anti-DDX46 (1:1000; Bethyl Laboratories A301-052A-T) used with Immuno Shot reagent 1 (Cosmo Bio), anti-HA rabbit (1:1000; Abm G166), anti-HA mouse (1:1000; Sigma-Aldrich H3663), and anti-ACTIN (1:2000; Sigma-Aldrich A2066).

### Minigene assays

The *ORAI2*, *ZNF91*, *MAP3K7*, and *TTI1* minigenes were cloned in our previous study (Zhang et al. 2019a). Minigene assays were performed as described (Zhang et al. 2022a). Briefly, HEK293T cells were transfected with 2 μg of expression plasmid DNA and a mixture of the four minigenes mentioned above (100 ng each) using Lipofectamine 2000 (Thermo Fisher Scientific). After 48 h, total RNA was extracted, treated with DNase I, and then reverse-transcribed using Maxima reverse transcriptase (Thermo Fisher Scientific) with oligo-dT primer and vector-specific reverse primer. PCR containing [α-^32^P] dCTP was performed with vector-specific forward primer and each of the minigene-specific reverse primers. PCR products were then resolved by 6% nondenaturing PAGE, and radioactive signals were detected by phosphorimaging (GE Healthcare). The primer sequences were listed in our previous study (Zhang et al. 2019a).

### Affinity purification using FLAG and GST tags

The small-scale affinity purification using FLAG and GST tags was performed as described (Zhang et al. 2022a), except with modifications to the NaCl concentrations in the buffers (see below). Briefly, HEK293T cells were transfected with 4 µg of expression plasmid DNA and split 1:4 on the next day, followed by 48 h of growth. Cells were lysed by incubation for 20 min at 4°C with lysis buffer (30 mM Tris-Cl at pH 7.4, 150 mM NaCl [except that 300 mM NaCl was used for Fig. 4], 1 mM EDTA, 0.5% Triton X-100, 10 mM sodium orthovanadate, 10 mM sodium fluoride) supplemented with protease inhibitor cocktail (Roche), PhosSTOP (Roche), and 200 µg of RNase A. After centrifugation, the cell extract (supernatant) was immunoprecipitated by incubation for 30 min at 4°C with 5 µg of anti-DYKDDDDK antibody (GenScript A00187) and then for 3 h with 50 µL of Pierce Protein A/G magnetic beads (Thermo Fisher Scientific). After four washes, proteins were eluted from the beads with elution buffer (30 mM Tris-Cl at pH 7.4, 150 mM NaCl [except that 300 mM NaCl was used for Fig. 4], 0.5% Triton X-100, 150 ng/μL 3× FLAG peptide) and then incubated with 40 µL of Glutathione Sepharose 4B beads (GE Healthcare) overnight at 4°C. After four washes, proteins were eluted using 1× SDS loading buffer.

### Knockdown experiments

Knockdown experiments were performed as described (Zhang et al. 2019a). Briefly, HEK293T cells were initially transfected with a final concentration of 10 nM siRNA and, after 24 h, transfected a second time with 10 nM siRNA using DharmaFECT 1 reagent (Dharmacon). After 24 h, total RNA and proteins were extracted from the transfected cells using TRIzol (Thermo Fisher Scientific). The sequences of the two siRNAs targeting *DDX46* were siDDX46-1 sense strand (5′-GAGGUAAAUGUGUUUCGAUdTdT-3′), siDDX46-1 antisense strand (5′-AUCGAAACACAUUUACCUCdTdT-3′), siDDX46-2 sense strand (5′-GCAGCUCUUGGUCUACAAGdTdT-3′), and siDDX46-2 antisense strand (5′-CUUGUAGACCAAGAGCUGCdTdT-3′). The sequences of the sense and antisense strands of the negative control siRNA (siC) were listed in our previous study (Zhang et al. 2019a). RT-PCR was performed as described (Zhang et al. 2019a). Briefly, total RNA was reverse-transcribed using oligo-dT primer and Maxima reverse transcriptase (Thermo Fisher Scientific), followed by PCR containing [α-^32^P] dCTP using the primers listed in our previous study (Zhang et al. 2019a). PCR products were resolved by 6% nondenaturing PAGE, and their radioactive signals were detected by a phosphor screen, which was then scanned using a Typhoon FLA 7000 imager (GE Healthcare).

### Protein expression, purification, and complex formation

Human SF3B complex and DHX15–SUGP1 were expressed individually in insect cells. SF3B1 (residues 453–1304), SF3B3 (residues 1–1217), SF3B5 (residues 1–86), and PHF5A (residues 1–98) were cloned into a pFastBac Serious-438 MacroBac vector. SF3B3 was fused with an N-terminal His6 and TEV protease cleavage site. DHX15 (residues 113–795) and SUGP1 (residues 433–645) were cloned into another pFastBac Serious-438 MacroBac vector. DHX15 was fused with an N-terminal His6, MBP, and TEV protease cleavage site. Two separate baculoviruses were generated by infecting Sf9 insect cells (*Spodoptera frugiperda*; Expression Systems). Protein complexes were expressed in Tni insect cells (*Trichoplusia ni*; Expression Systems) for 2 d at 27°C.

For protein purification, the DHX15–SUGP1 complex was obtained by following the previous protocol (Zhang et al. 2022a). The SF3B complex cell pellets were resuspended in lysis buffer containing 20 mM HEPES (pH 7.9), 600 mM KCl, 20 mM imidazole, 10% (v/v) glycerol, and 10 mM β-mercaptoethanol supplemented with SigmaFast EDTA-free protease inhibitor cocktail (Sigma-Aldrich). After sonication, the cell-free lysate was obtained by centrifugation and then mixed with pre-equilibrated Ni-NTA beads (Qiagen) for 1 h at 4°C. The nickel beads were washed with at least five column volumes of lysis buffer, and then the target proteins were eluted with a buffer containing 20 mM HEPES (pH 7.9), 600 mM KCl, 250 mM imidazole, 10% (v/v) glycerol, and 10 mM β-mercaptoethanol. The fractions containing high absorbance at 280 nm were pooled and injected onto a HiLoad 16/600 Superdex 200 preparation-grade gel filtration column (Cytiva) that was equilibrated with 20 mM HEPES (pH 7.9), 300 mM KCl, 5% (v/v) glycerol, and 5 mM DTT. Fractions with the target proteins were pooled and concentrated. Both protein complexes were flash-frozen in liquid nitrogen and stored at −80°C.

Purified SF3B complex and DHX15 (113–795)–SUGP1 (433–645) heterodimer were mixed at a molar ratio of 1:5, incubated overnight at 4°C, and injected onto the Superose 6 Increase 10/300 gel filtration column (Cytiva) pre-equilibrated with 20 mM HEPES (pH 7.9), 300 mM KCl, 5% (v/v) glycerol, and 5 mM DTT. Fractions containing the complex were pooled, concentrated, and rerun on the Superose 6 column. Fractions with high absorbance at 280 nm were collected, concentrated, and run on an SDS-PAGE gel to verify the existence of the complex.

### Quantification and statistical analysis

Radioactive signals of ^32^P RT-PCR products in minigene assays were detected by phosphorimaging and then quantified using ImageQuant (Molecular Dynamics). Statistical differences in PSI values were determined by the unpaired, two-tailed, and unequal variance *t*-tests using Excel (Microsoft).

### Data availability

Predicted structures reported in this study were deposited in ModelArchive (https://www.modelarchive.org) with the accession codes ma-cojyq (heterodimer of SF3B1 HEAT domain and SUGP1) and ma-s75h3 (heterotrimer of SF3B1 HEAT domain, SUGP1, and DHX15).

## Supplementary Material

Supplement 1

Supplement 2

Supplement 3

Supplement 4

Supplement 5

Supplement 6

## References

[GAD351154ZHAC1] Agafonov DE, Kastner B, Dybkov O, Hofele RV, Liu WT, Urlaub H, Lührmann R, Stark H. 2016. Molecular architecture of the human U4/U6.U5 tri-snRNP. Science 351: 1416–1420. 10.1126/science.aad2085 26912367

[GAD351154ZHAC2] Alsafadi S, Houy A, Battistella A, Popova T, Wassef M, Henry E, Tirode F, Constantinou A, Piperno-Neumann S, Roman-Roman S, 2016. Cancer-associated SF3B1 mutations affect alternative splicing by promoting alternative branchpoint usage. Nat Commun 7: 10615. 10.1038/ncomms10615 26842708 PMC4743009

[GAD351154ZHAC3] Alsafadi S, Dayot S, Tarin M, Houy A, Bellanger D, Cornella M, Wassef M, Waterfall JJ, Lehnert E, Roman-Roman S, 2021. Genetic alterations of SUGP1 mimic mutant-SF3B1 splice pattern in lung adenocarcinoma and other cancers. Oncogene 40: 85–96. 10.1038/s41388-020-01507-5 33057152 PMC7790757

[GAD351154ZHAC4] Berman HM, Westbrook J, Feng Z, Gilliland G, Bhat TN, Weissig H, Shindyalov IN, Bourne PE. 2000. The protein data bank. Nucleic Acids Res 28: 235–242. 10.1093/nar/28.1.235 10592235 PMC102472

[GAD351154ZHAC5] Bertram K, Agafonov DE, Dybkov O, Haselbach D, Leelaram MN, Will CL, Urlaub H, Kastner B, Lührmann R, Stark H. 2017a. Cryo-EM structure of a pre-catalytic human spliceosome primed for activation. Cell 170: 701–713.e11. 10.1016/j.cell.2017.07.011 28781166

[GAD351154ZHAC6] Bertram K, Agafonov DE, Liu WT, Dybkov O, Will CL, Hartmuth K, Urlaub H, Kastner B, Stark H, Lührmann R. 2017b. Cryo-EM structure of a human spliceosome activated for step 2 of splicing. Nature 542: 318–323. 10.1038/nature21079 28076346

[GAD351154ZHAC7] Beusch I, Rao B, Studer MK, Luhovska T, Šukytė V, Lei S, Oses-Prieto J, SeGraves E, Burlingame A, Jonas S, 2023. Targeted high throughput mutagenesis of the human spliceosome reveals its in vivo operating principles. Mol Cell 83: 2578–2594.e9. 10.1016/j.molcel.2023.06.003 37402368 PMC10484158

[GAD351154ZHAC8] Biankin AV, Waddell N, Kassahn KS, Gingras MC, Muthuswamy LB, Johns AL, Miller DK, Wilson PJ, Patch AM, Wu J, 2012. Pancreatic cancer genomes reveal aberrations in axon guidance pathway genes. Nature 491: 399–405. 10.1038/nature11547 23103869 PMC3530898

[GAD351154ZHAC9] Choi IY, Ling JP, Zhang J, Helmenstine E, Walter W, Bergman RE, Philippe C, Manley JL, Rouault-Pierre K, Li B, 2023. The E592K variant of SF3B1 creates unique RNA missplicing and associates with high-risk MDS without ring sideroblasts. Res Sq. 10.21203/rs.3.rs-2802265/v1 PMC11331715 38759096

[GAD351154ZHAC10] Cretu C, Schmitzová J, Ponce-Salvatierra A, Dybkov O, De Laurentiis EI, Sharma K, Will CL, Urlaub H, Lührmann R, Pena V. 2016. Molecular architecture of SF3b and structural consequences of its cancer-related mutations. Mol Cell 64: 307–319. 10.1016/j.molcel.2016.08.036 27720643

[GAD351154ZHAC11] Crisci A, Raleff F, Bagdiul I, Raabe M, Urlaub H, Rain JC, Krämer A. 2015. Mammalian splicing factor SF1 interacts with SURP domains of U2 snRNP-associated proteins. Nucleic Acids Res 43: 10456–10473. 10.1093/nar/gkv952 26420826 PMC4666396

[GAD351154ZHAC12] Darman RB, Seiler M, Agrawal AA, Lim KH, Peng S, Aird D, Bailey SL, Bhavsar EB, Chan B, Colla S, 2015. Cancer-associated SF3B1 hotspot mutations induce cryptic 3′ splice site selection through use of a different branch point. Cell Rep 13: 1033–1045. 10.1016/j.celrep.2015.09.053 26565915

[GAD351154ZHAC13] DeBoever C, Ghia EM, Shepard PJ, Rassenti L, Barrett CL, Jepsen K, Jamieson CH, Carson D, Kipps TJ, Frazer KA. 2015. Transcriptome sequencing reveals potential mechanism of cryptic 3′ splice site selection in SF3B1-mutated cancers. PLoS Comput Biol 11: e1004105. 10.1371/journal.pcbi.1004105 25768983 PMC4358997

[GAD351154ZHAC14] Ellis MJ, Ding L, Shen D, Luo J, Suman VJ, Wallis JW, Van Tine BA, Hoog J, Goiffon RJ, Goldstein TC, 2012. Whole-genome analysis informs breast cancer response to aromatase inhibition. Nature 486: 353–360. 10.1038/nature11143 22722193 PMC3383766

[GAD351154ZHAC15] Evans R, O'Neill M, Pritzel A, Antropova N, Senior A, Green T, Žídek A, Bates R, Blackwell S, Yim J. 2022. Protein complex prediction with AlphaFold-Multimer. bioRxiv 10.1101/2021.10.04.463034

[GAD351154ZHAC16] Feng Q, Krick K, Chu J, Burge CB. 2023. Splicing quality control mediated by DHX15 and its G-patch activator SUGP1. Cell Rep 42: 113223. 10.1016/j.celrep.2023.113223 37805921 PMC10842378

[GAD351154ZHAC17] Fourmann JB, Dybkov O, Agafonov DE, Tauchert MJ, Urlaub H, Ficner R, Fabrizio P, Lührmann R. 2016. The target of the DEAH-box NTP triphosphatase Prp43 in *Saccharomyces cerevisiae* spliceosomes is the U2 snRNP–intron interaction. Elife 5: e15564. 10.7554/eLife.15564 27115347 PMC4866824

[GAD351154ZHAC18] Haferlach T, Nagata Y, Grossmann V, Okuno Y, Bacher U, Nagae G, Schnittger S, Sanada M, Kon A, Alpermann T, 2014. Landscape of genetic lesions in 944 patients with myelodysplastic syndromes. Leukemia 28: 241–247. 10.1038/leu.2013.336 24220272 PMC3918868

[GAD351154ZHAC19] Harbour JW, Roberson ED, Anbunathan H, Onken MD, Worley LA, Bowcock AM. 2013. Recurrent mutations at codon 625 of the splicing factor SF3B1 in uveal melanoma. Nat Genet 45: 133–135. 10.1038/ng.2523 23313955 PMC3789378

[GAD351154ZHAC20] Haselbach D, Komarov I, Agafonov DE, Hartmuth K, Graf B, Dybkov O, Urlaub H, Kastner B, Lührmann R, Stark H. 2018. Structure and conformational dynamics of the human spliceosomal B^act^ complex. Cell 172: 454–464.e11. 10.1016/j.cell.2018.01.010 29361316

[GAD351154ZHAC21] Hegele A, Kamburov A, Grossmann A, Sourlis C, Wowro S, Weimann M, Will CL, Pena V, Lührmann R, Stelzl U. 2012. Dynamic protein-protein interaction wiring of the human spliceosome. Mol Cell 45: 567–580. 10.1016/j.molcel.2011.12.034 22365833

[GAD351154ZHAC22] Hintzsche JD, Gorden NT, Amato CM, Kim J, Wuensch KE, Robinson SE, Applegate AJ, Couts KL, Medina TM, Wells KR, 2017. Whole-exome sequencing identifies recurrent SF3B1 R625 mutation and comutation of NF1 and KIT in mucosal melanoma. Melanoma Res 27: 189–199. 10.1097/CMR.0000000000000345 28296713 PMC5470740

[GAD351154ZHAC23] Ho SN, Hunt HD, Horton RM, Pullen JK, Pease LR. 1989. Site-directed mutagenesis by overlap extension using the polymerase chain reaction. Gene 77: 51–59. 10.1016/0378-1119(89)90358-2 2744487

[GAD351154ZHAC24] Inoue D, Chew GL, Liu B, Michel BC, Pangallo J, D'Avino AR, Hitchman T, North K, Lee SC, Bitner L, 2019. Spliceosomal disruption of the non-canonical BAF complex in cancer. Nature 574: 432–436. 10.1038/s41586-019-1646-9 31597964 PMC6858563

[GAD351154ZHAC25] Jumper J, Evans R, Pritzel A, Green T, Figurnov M, Ronneberger O, Tunyasuvunakool K, Bates R, Žídek A, Potapenko A, 2021. Highly accurate protein structure prediction with AlphaFold. Nature 596: 583–589. 10.1038/s41586-021-03819-2 34265844 PMC8371605

[GAD351154ZHAC26] Lieu YK, Liu Z, Ali AM, Wei X, Penson A, Zhang J, An X, Rabadan R, Raza A, Manley JL, 2022. SF3B1 mutant-induced missplicing of MAP3K7 causes anemia in myelodysplastic syndromes. Proc Natl Acad Sci 119: e2111703119. 10.1073/pnas.2111703119 34930825 PMC8740767

[GAD351154ZHAC27] Liu Z, Zhang J, Sun Y, Perea-Chamblee TE, Manley JL, Rabadan R. 2020. Pan-cancer analysis identifies mutations in *SUGP1* that recapitulate mutant SF3B1 splicing dysregulation. Proc Natl Acad Sci 117: 10305–10312. 10.1073/pnas.1922622117 32332164 PMC7229667

[GAD351154ZHAC28] Maul-Newby HM, Amorello AN, Sharma T, Kim JH, Modena MS, Prichard BE, Jurica MS. 2022. A model for DHX15 mediated disassembly of A-complex spliceosomes. RNA 28: 583–595. 10.1261/rna.078977.121 35046126 PMC8925973

[GAD351154ZHAC29] Murakami K, Nakano K, Shimizu T, Ohto U. 2017. The crystal structure of human DEAH-box RNA helicase 15 reveals a domain organization of the mammalian DEAH/RHA family. Acta Crystallogr F Struct Biol Commun 73: 347–355. 10.1107/S2053230X17007336 28580923 PMC5458392

[GAD351154ZHAC30] Papaemmanuil E, Cazzola M, Boultwood J, Malcovati L, Vyas P, Bowen D, Pellagatti A, Wainscoat JS, Hellstrom-Lindberg E, Gambacorti-Passerini C, 2011. Somatic *SF3B1* mutation in myelodysplasia with ring sideroblasts. N Engl J Med 365: 1384–1395. 10.1056/NEJMoa1103283 21995386 PMC3322589

[GAD351154ZHAC31] Quesada V, Ramsay AJ, Lopez-Otin C. 2012. Chronic lymphocytic leukemia with *SF3B1* mutation. N Engl J Med 366: 2530. 10.1056/NEJMc1204033 22738114

[GAD351154ZHAC32] Rodrigues KS, Petroski LP, Utumi PH, Ferrasa A, Herai RH. 2023. IARA: a complete and curated atlas of the biogenesis of spliceosome machinery during RNA splicing. Life Sci Alliance 6: e202201593. 10.26508/lsa.202201593 36609432 PMC9834665

[GAD351154ZHAC33] Sampson ND, Hewitt JE. 2003. SF4 and SFRS14, two related putative splicing factors on human chromosome 19p13.11. Gene 305: 91–100. 10.1016/s0378-1119(02)01230-1 12594045

[GAD351154ZHAC34] Seiler M, Peng S, Agrawal AA, Palacino J, Teng T, Zhu P, Smith PG, Cancer Genome Atlas Research N, Buonamici S, Yu L. 2018. Somatic mutational landscape of splicing factor genes and their functional consequences across 33 cancer types. Cell Rep 23: 282–296.e4. 10.1016/j.celrep.2018.01.088 29617667 PMC5933844

[GAD351154ZHAC35] Studer MK, Ivanović L, Weber ME, Marti S, Jonas S. 2020. Structural basis for DEAH-helicase activation by G-patch proteins. Proc Natl Acad Sci 117: 7159–7170. 10.1073/pnas.1913880117 32179686 PMC7132122

[GAD351154ZHAC36] Tholen J, Razew M, Weis F, Galej WP. 2022. Structural basis of branch site recognition by the human spliceosome. Science 375: 50–57. 10.1126/science.abm4245 34822310 PMC7614990

[GAD351154ZHAC37] UniProt Consortium. 2023. UniProt: the Universal Protein Knowledgebase in 2023. Nucleic Acids Res 51: D523–D531. 10.1093/nar/gkac1052 36408920 PMC9825514

[GAD351154ZHAC38] Wahl MC, Will CL, Lührmann R. 2009. The spliceosome: design principles of a dynamic RNP machine. Cell 136: 701–718. 10.1016/j.cell.2009.02.009 19239890

[GAD351154ZHAC39] Wang L, Lawrence MS, Wan Y, Stojanov P, Sougnez C, Stevenson K, Werner L, Sivachenko A, DeLuca DS, Zhang L, 2011. *SF3B1* and other novel cancer genes in chronic lymphocytic leukemia. N Engl J Med 365: 2497–2506. 10.1056/NEJMoa1109016 22150006 PMC3685413

[GAD351154ZHAC40] Xue LC, Rodrigues JP, Kastritis PL, Bonvin AM, Vangone A. 2016. PRODIGY: a web server for predicting the binding affinity of protein–protein complexes. Bioinformatics 32: 3676–3678. 10.1093/bioinformatics/btw514 27503228

[GAD351154ZHAC41] Yoshida K, Ogawa S. 2014. Splicing factor mutations and cancer. Wiley Interdiscip Rev RNA 5: 445–459. 10.1002/wrna.1222 24523246

[GAD351154ZHAC42] Yoshida K, Sanada M, Shiraishi Y, Nowak D, Nagata Y, Yamamoto R, Sato Y, Sato-Otsubo A, Kon A, Nagasaki M, 2011. Frequent pathway mutations of splicing machinery in myelodysplasia. Nature 478: 64–69. 10.1038/nature10496 21909114

[GAD351154ZHAC43] Zhan X, Yan C, Zhang X, Lei J, Shi Y. 2018. Structures of the human pre-catalytic spliceosome and its precursor spliceosome. Cell Res 28: 1129–1140. 10.1038/s41422-018-0094-7 30315277 PMC6274647

[GAD351154ZHAC44] Zhang X, Yan C, Hang J, Finci LI, Lei J, Shi Y. 2017. An atomic structure of the human spliceosome. Cell 169: 918–929.e14. 10.1016/j.cell.2017.04.033 28502770

[GAD351154ZHAC45] Zhang X, Yan C, Zhan X, Li L, Lei J, Shi Y. 2018. Structure of the human activated spliceosome in three conformational states. Cell Res 28: 307–322. 10.1038/cr.2018.14 29360106 PMC5835773

[GAD351154ZHAC46] Zhang J, Ali AM, Lieu YK, Liu Z, Gao J, Rabadan R, Raza A, Mukherjee S, Manley JL. 2019a. Disease-causing mutations in SF3B1 alter splicing by disrupting interaction with SUGP1. Mol Cell 76: 82–95.e7. 10.1016/j.molcel.2019.07.017 31474574 PMC7065273

[GAD351154ZHAC47] Zhang X, Zhan X, Yan C, Zhang W, Liu D, Lei J, Shi Y. 2019b. Structures of the human spliceosomes before and after release of the ligated exon. Cell Res 29: 274–285. 10.1038/s41422-019-0143-x 30728453 PMC6461851

[GAD351154ZHAC48] Zhang Z, Will CL, Bertram K, Dybkov O, Hartmuth K, Agafonov DE, Hofele R, Urlaub H, Kastner B, Lührmann R, 2020. Molecular architecture of the human 17S U2 snRNP. Nature 583: 310–313. 10.1038/s41586-020-2344-3 32494006

[GAD351154ZHAC49] Zhang J, Huang J, Xu K, Xing P, Huang Y, Liu Z, Tong L, Manley JL. 2022a. DHX15 is involved in SUGP1-mediated RNA missplicing by mutant SF3B1 in cancer. Proc Natl Acad Sci 119: e2216712119. 10.1073/pnas.2216712119 36459648 PMC9894173

[GAD351154ZHAC50] Zhang X, Zhan X, Bian T, Yang F, Li P, Lu Y, Xing Z, Zhang QC, Shi Y. 2022b. Structural insights into branch site proofreading by human spliceosome. bioRxiv 10.1101/2022.11.07.515429 38196034

