# Supplementary material for: Characterization of the SF3B1–SUGP1 interface reveals how numerous cancer mutations cause mRNA missplicing
Source: Genes Dev. 2023 Nov-Dec;37(21-24):968–83. doi: 10.1101/gad.351154.123 (PMC10760632; doi:10.1101/gad.351154.123)
Supplement: Supplement 1 [file Supplemental_Fig_S1.pdf]

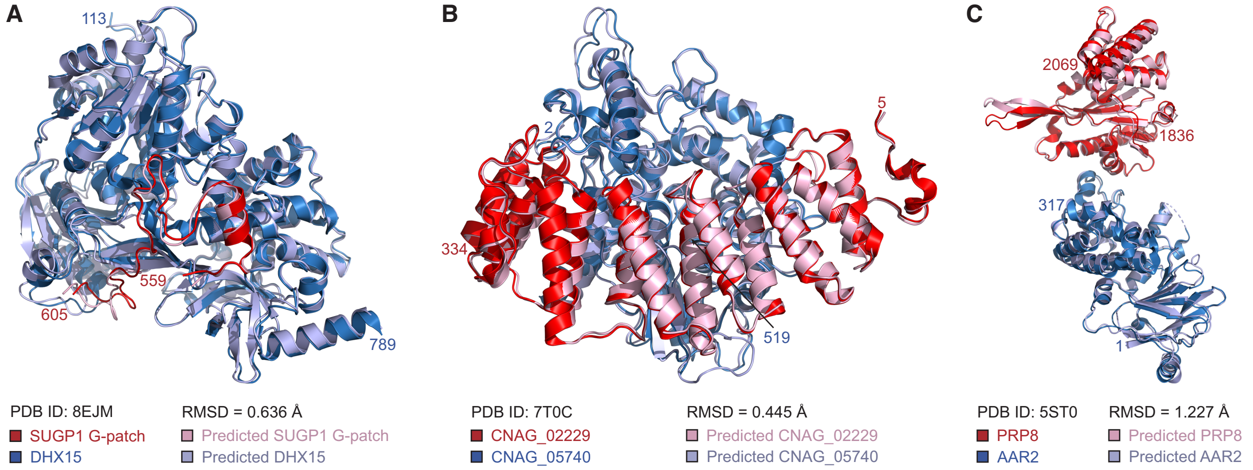

Supplemental Figure S1. Evaluation of AlphaFold-Multimer on three known complexes. (*A–C*) Alignment of AlphaFold-Multimer predicted structures and crystal structures of complexes of DHX15 (113–789) with SUGP1 G-patch (559–605) (*A*), of CNAG_02229 (5–334) with CNAG_05740 (2–519) (*B*), and of PRP8 (1836–2069) and AAR2 (1–317) (*C*). Residues from the expression tags (and/or the cloning vectors) attached to the crystallized proteins were also included in the AlphaFold-Multimer predictions. Although their presence or absence did not affect calculations, they were removed in the structure alignments and RMSD calculations shown in the panels.
