# Supplementary material for: Characterization of the SF3B1–SUGP1 interface reveals how numerous cancer mutations cause mRNA missplicing
Source: Genes Dev. 2023 Nov-Dec;37(21-24):968–83. doi: 10.1101/gad.351154.123 (PMC10760632; doi:10.1101/gad.351154.123)
Supplement: Supplement 2 [file Supplemental_Fig_S2.pdf]

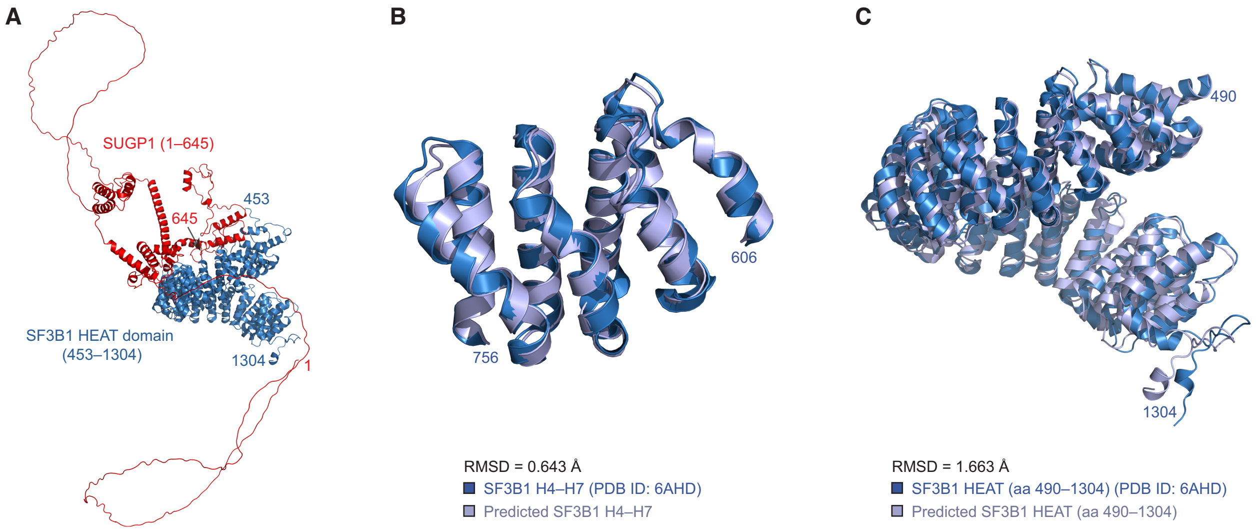

Supplemental Figure S2. Predicted structure of the SF3B1 HEAT domain-SUGP1 heterodimer. (*A*) Schematic drawing of the predicted structure of the SF3B1-SUGP1 heterodimer. The SF3B1 HEAT domain (453–1304) is shown in blue, and the full-length SUGP1 (1–645) in red. (*B*,*C*) Alignment of the predicted structure with the cryo-EM structure of SF3B1 H4–H7 (*B*), or of SF3B1 HEAT domain residues 490–1304 (*C*).
