# Supplementary material for: Characterization of the SF3B1–SUGP1 interface reveals how numerous cancer mutations cause mRNA missplicing
Source: Genes Dev. 2023 Nov-Dec;37(21-24):968–83. doi: 10.1101/gad.351154.123 (PMC10760632; doi:10.1101/gad.351154.123)
Supplement: Supplement 4 [file Supplemental_Fig_S4.pdf]

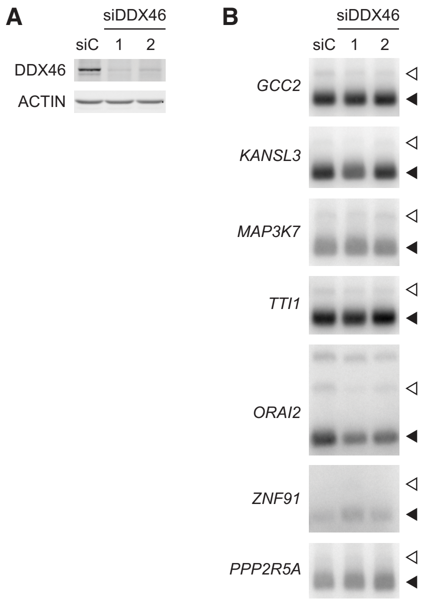

Supplemental Figure S4. DDX46 knockdown does not induce cryptic 3′ss usage in any of the top targets of mutant SF3B1. (*A*) HEK293T cells were transfected with a negative control siRNA (siC) or one of two independent siRNAs targeting DDX46, followed by western blotting. (*B*) ^{32}P RT-PCR products of the cryptic (open arrowheads) and canonical (solid arrowheads) 3′ss of the indicated genes in HEK293T cells in (*A*).
