# Supplementary material for: Characterization of the SF3B1–SUGP1 interface reveals how numerous cancer mutations cause mRNA missplicing
Source: Genes Dev. 2023 Nov-Dec;37(21-24):968–83. doi: 10.1101/gad.351154.123 (PMC10760632; doi:10.1101/gad.351154.123)
Supplement: Supplement 3 [file Supplemental_Fig_S3.pdf]

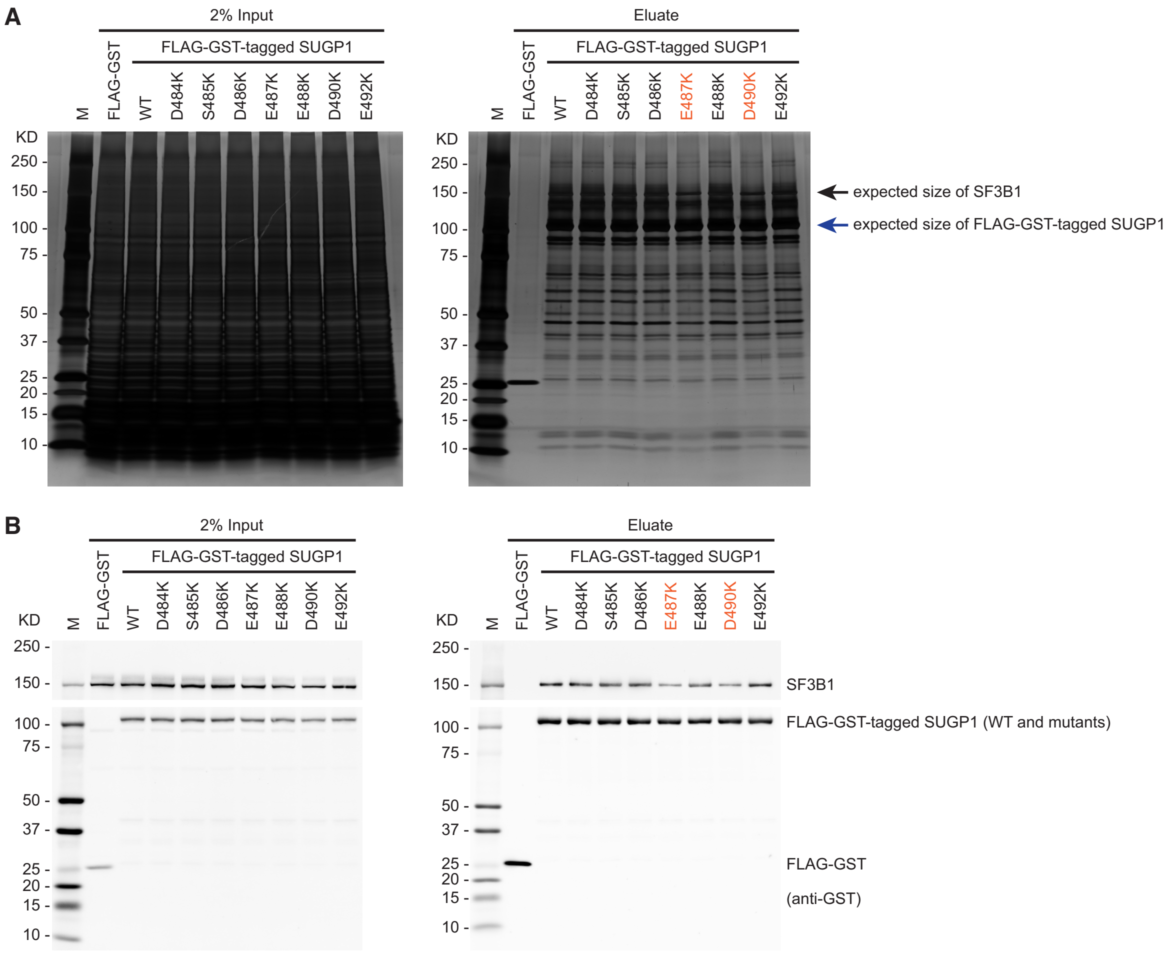

Supplemental Figure S3. Effects of single mutations in the negatively charged region on the SUGP1-SF3B1 interaction. (*A*,*B*) HEK293T cells were transfected with expression plasmid for FLAG-GST tandem tags or FLAG-GST-tagged SUGP1 (WT or one of its mutants as indicated), followed by affinity purification using FLAG and GST tags. Purified proteins were resolved by SDS-PAGE, followed by silver staining (*A*) or western blotting (*B*). M, Precision Plus Protein marker (Bio-Rad).
